# Capillary‐Force‐Assisted Optical Tuning of Coupled Plasmons

**DOI:** 10.1002/adma.201503292

**Published:** 2015-09-23

**Authors:** Tao Ding, Jan Mertens, Daniel O. Sigle, Jeremy J. Baumberg

**Affiliations:** ^1^ Nanophotonics CentreCavendish LaboratoryUniversity of CambridgeCambridgeCB3 0HEUK

**Keywords:** polymer spacer layers, plasmon resonance, gold, nanogaps, spectra

## Abstract

**An ultrathin (few nanometer) polymer spacer layer** is softened by local optical heating and restructured by strong capillary forces, which increase the gap between the plasmonic metal components. This results in a continuous blue‐shift of the coupled plasmon from near infrared to visible with a tuning range of >150 nm that can be tightly controlled by adjusting either irradiation time or power.

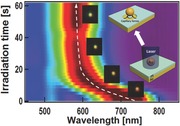

Plasmons, formed by the collective oscillation of free electrons inside metals at optical frequencies, have been vigorously investigated in both fundamental studies and practical applications, including surface enhanced spectroscopies, photocatalysis, solar cells, and photothermal therapies.^1^, ^2^, ^3^, ^4^ In most of these, tailoring the plasmon resonance to a desired spectral position is crucial to overlap plasmonic resonances with appropriate electronic or molecular transitions. Significant efforts have been devoted to creating tuneable plasmonic systems, eliciting several approaches such as wet chemistry modification,^5^ top‐down fabrication,^6^ dynamic self‐assembly and disassembly,^7^, ^8^, ^9^, ^10^ electrical tuning,^11^ and mechanical stretching.^12^ The basic general principle is to change the size, shape, surroundings, or distance between different plasmonic components which tunes the surface plasmon resonance. However, these methods either suffer from a lack of in‐situ tuning and on‐demand control, from small tuning ranges (<50 nm), or are expensive and complicated fabrication procedures are required.

Inducing such tuning by light would have unique advantages of on‐demand and remote control, enabling for instance trimming an entire array to have the exact plasmon resonance desired.^13^ Although irradiation has been used to restructure the shape of Ag nanoparticles (NP) into nanoprisms, such bi‐state switching is irreversible, restricted to jump tuning, and has only a small tuning range.^14^, ^15^ When plasmonic elements are spaced within a few nm, the extreme optical confinement brings great sensitivity to the gap contents, which can be harnessed for tuning.^16^ We recently applied laser irradiation to tune plasmon coupling between Au NPs spaced above a Au mirror by sub‐nanometer gaps,^17^, ^18^ but the IR tuning range is rather limited (<50 nm) and outside the visible. A second aspect to such tuning is that extremely small volumes ≈1 nm^3^ control the spectral position, thus interrogating materials on the nanoscale such as polymers that are typically out of reach of electron microscopy.

Here, we demonstrate how low power light can be used for broadband optical tuning of plasmonic resonances. Ultrathin polymer spacers (≈4 nm spin coated, Figure S1, Supporting Information) are utilized in the NP on mirror (NPoM) geometry to separate each Au NP from the planar Au mirror underneath. Such spacer thicknesses (calibrated by ellipsometry) result in an intense coupled plasmonic mode of the NPoM around 730 nm, resulting from the interaction between the dipole induced in the NP and its image dipole in the mirror, forming a longitudinal mode with a dipole perpendicular to the surface. This geometry can generate field enhancements of 10^3^.^19^ By laser irradiating individual NPoMs with blue light (447 nm, 0.5 mW), we observe a gradual blue‐shift of the longitudinal plasmon mode from 730 to 580 nm (**Figure** **1**a) while the scattering color of the Au NP changes from red to yellow to green (inset of Figure 1a). The plasmon resonance continuously shifts to the blue over the first 20 s of irradiation (Figure 1b), and eventually saturates at 584 nm after 30 s of irradiation. Irradiating other Au NPs shows similar trends (Figure S2, Supporting Information) although the starting wavelength and blue‐shift rate vary slightly, mainly due to the slightly variable size of AuNPs or spacer thickness. Control experiments using the same irradiation conditions but with spacers of deposited silica films exhibit no shift (Figure S3, Supporting Information), showing that the polymer layer is critical to obtain tuning. Scattering spectra are recorded over long intervals after repeated 2 s pulses of laser irradiation followed by 40 s relaxation with the laser off (Figure S4, Supporting Information) demonstrating that the resonance peak wavelength (Figure 1c) can be progressively blue‐shifted in a discrete fashion. Slight subsequent red‐shifts are observed each time the laser is turned off. The morphology of Au NPs before and after irradiation (Figure 1d) show no apparent change (see also Figure S5, Supporting Information), excluding significant size/shape changes of the NP contributing to any blue‐shift. However, some changes are seen in the polymer layer localized underneath each Au NP (Figure 1f, white arrow).

**Figure 1:**
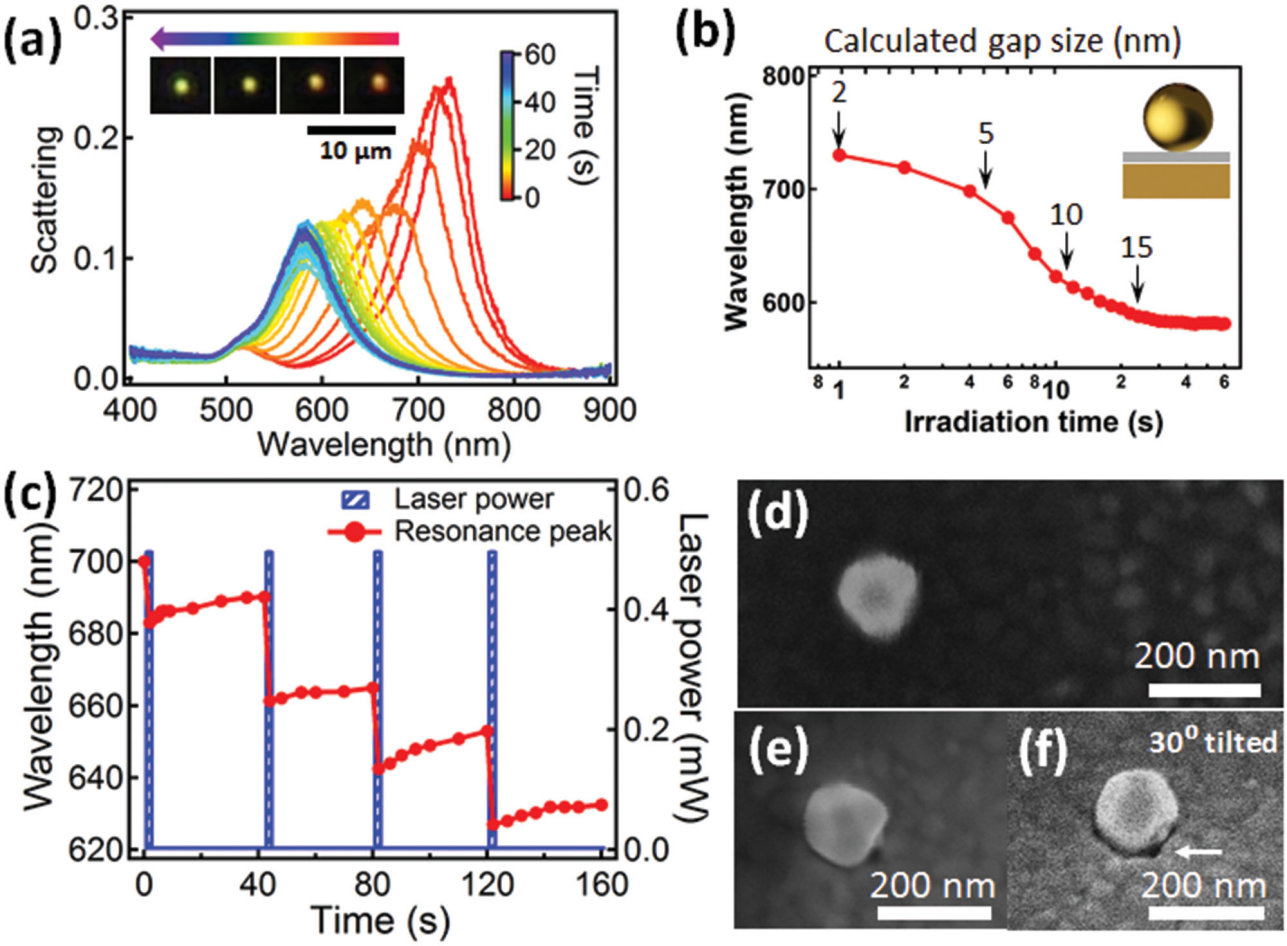
Nanoparticle on mirror (NPoM) under laser irradiation (447 nm, 0.5 mW) with 4 nm PS spacer layer. a) Scattering spectra at increasing irradiation times, inset shows color change of Au NPoMs over time. b) Extracted plasmon wavelength versus irradiation time, together with calculated gap size. Inset: NPoM with polymer spacer (gray). c) Resonance tuning after repeated irradiation pulses followed by relaxation. d–f) Scanning electron microscope (SEM) images of Au NPoM before and after irradiation, f) is a 30° tilted view, white arrow indicates local change in polymer.

In the following, we answer the question of what causes the blue‐shift, as well as why there is a red‐shift when the laser is turned off and how irradiation affects the polymer layer. A blue‐shift of the longitudinal plasmon mode in such Au NPoM constructs is either related to an increase of the gap size,^20^ a decrease of refractive index, or an increase in the conductivity of the gap material.^21^ To investigate the origin of the resonance shifts, surface enhanced Raman scattering (SERS) of individual NPoMs are recorded (Figure S6a, Supporting Information). Changes in the spacer refractive index or spacer conductivity should affect the SERS spectrum because it would reveal modifications in the chemical makeup of the polymer. For polystyrene (PS) spacers, the main Raman resonances around 1000 cm^−1^ (from the breathing mode of the aromatic ring) are unaltered throughout the irradiation process, implying that the chemical state of the PS remains unchanged. This implies that the conductivity remains constant throughout irradiation, and the refractive index of the PS is not affected (as it would have to change unfeasibly far in order to match the observed blue‐shifts). In turn, this suggests that the gap size increases during laser irradiation.

To support this hypothesis, we directly visualize the polymer underneath each NP after irradiation. To achieve this, we switch to Ag NPs which can be selectively removed with NH_3_ solution after irradiation, revealing changes of the polymer layer underneath each particle (**Figure** **2**a–c). The laser spot (white circle, which shows higher scanning electron microscope (SEM) contrast after irradiation) encompasses two Ag NPs which have been irradiated. After etching in NH_3_ solution for 2 h, Ag NPs are removed from the substrate, revealing laser‐induced redistribution of the polymer underneath. Tilted views further confirm the modified morphology of the polymer films giving small bumps under the irradiated NPs (Figure 2c). No such changes are seen for Ag NPs outside this spot that have not been irradiated. Simulations using the boundary element method show that the longitudinal mode blue‐shifts when the gap size increases (Figure 2d,e) and imply that in experiments the separation between NP and substrate increases from 4 ± 0.5 to 15 ± 3 nm (Figures 1b and 2e).

**Figure 2:**
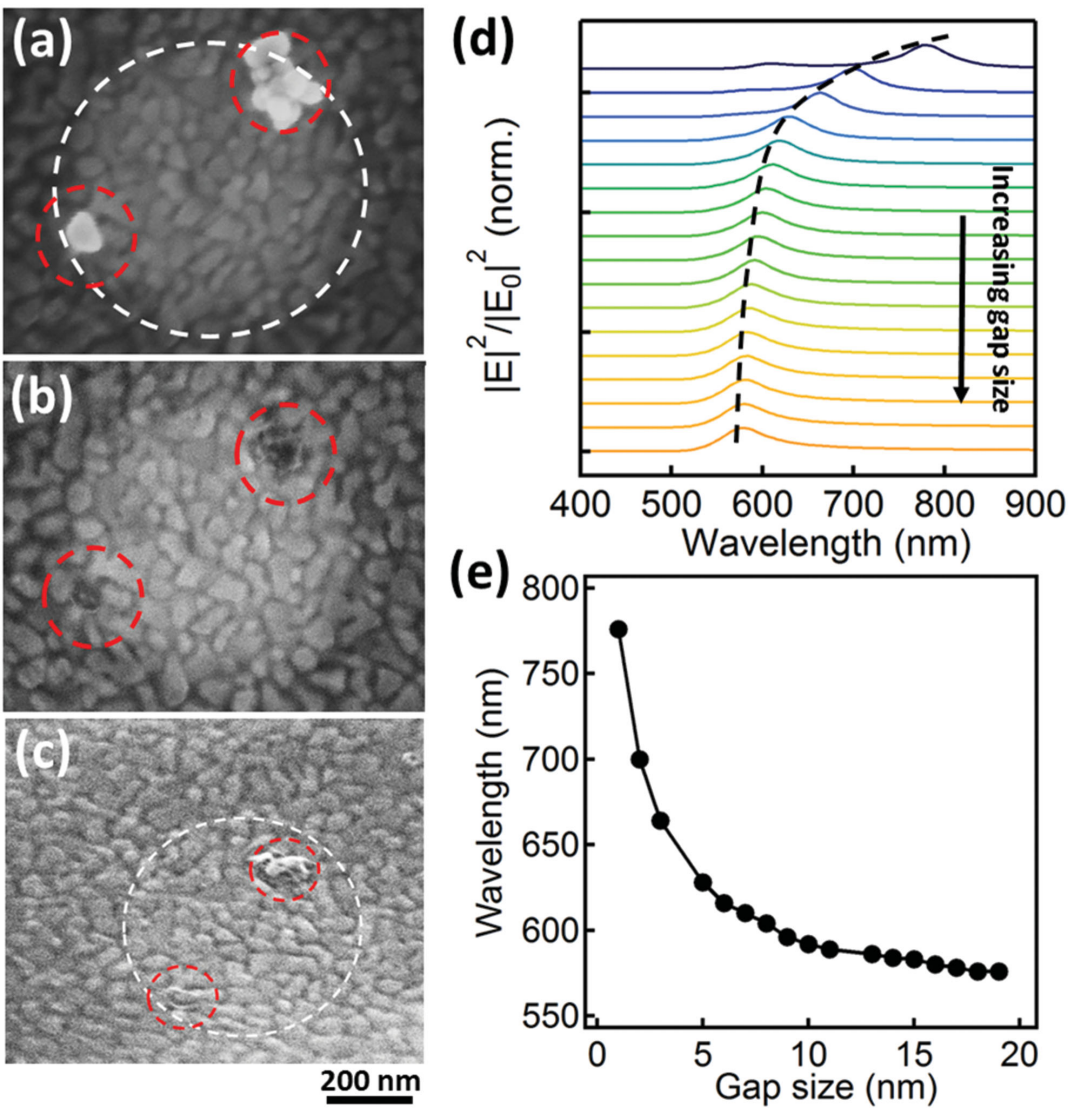
SEM images of irradiated Ag NPs on PS on Au, a) before, and b,c) after dissolving Ag NPs away. Laser irradiated spot (white dashed) shows irradiated (red dashed) Ag NPs, with (c) 40° tilted to show polymer bumps in (b). d) Simulated scattering spectra of Au NPoMs with increasing PS spacer width from 1–19 nm in 1 nm steps. e) Simulated resonance wavelength versus gap size.

To understand how laser irradiation can change the polymer layer sandwiched between Au NP and substrate, we first compare with global thermal annealing of the entire sample (so all NPoMs are affected simultaneously). In all cases we see the same typical behavior (**Figure** **3**) with first a gradual change of Au NP scattering color as the temperature increases up to 150 °C (Figure 3a). The coupled plasmon mode initially slightly red‐shifts by 8 nm (Figure 3b,c), likely due to a small decrease in separation between Au NP and mirror. Such a change can take place because the AuNP squashes slightly into the PS layer when the polymer softens above *T*_g_. However, when the temperature reaches 120 °C, the plasmon rapidly blue‐shifts by 70 nm reaching λ=710 nm at 150 °C. Cooling the sample back to 70 °C leads to another small red‐shift of 9 nm, eventually stabilizing at 718 nm at room temperature (Figure 3c). Well above *T*_g_ the PS is more like a viscous fluid rather than a rigid spacer. Because of the difference in surface energies between Au (8.8 N m^−1^)^22^ and PS (40 mN m^−1^), surface tension reshapes the PS fluid meniscus between Au NP and Au substrate, so that the strong capillary forces form a polymer bulge (**Figure** **4**). The distance between Au NP and Au mirror thus increases, creating the observed blue‐shift. When the PS temperature drops again below *T*_g_, the bulge shrinks only slightly, bringing the NP only slightly closer to the Au surface again and resulting in the observed slight red‐shift after cooling (Figures 1c and 3c).

**Figure 3:**
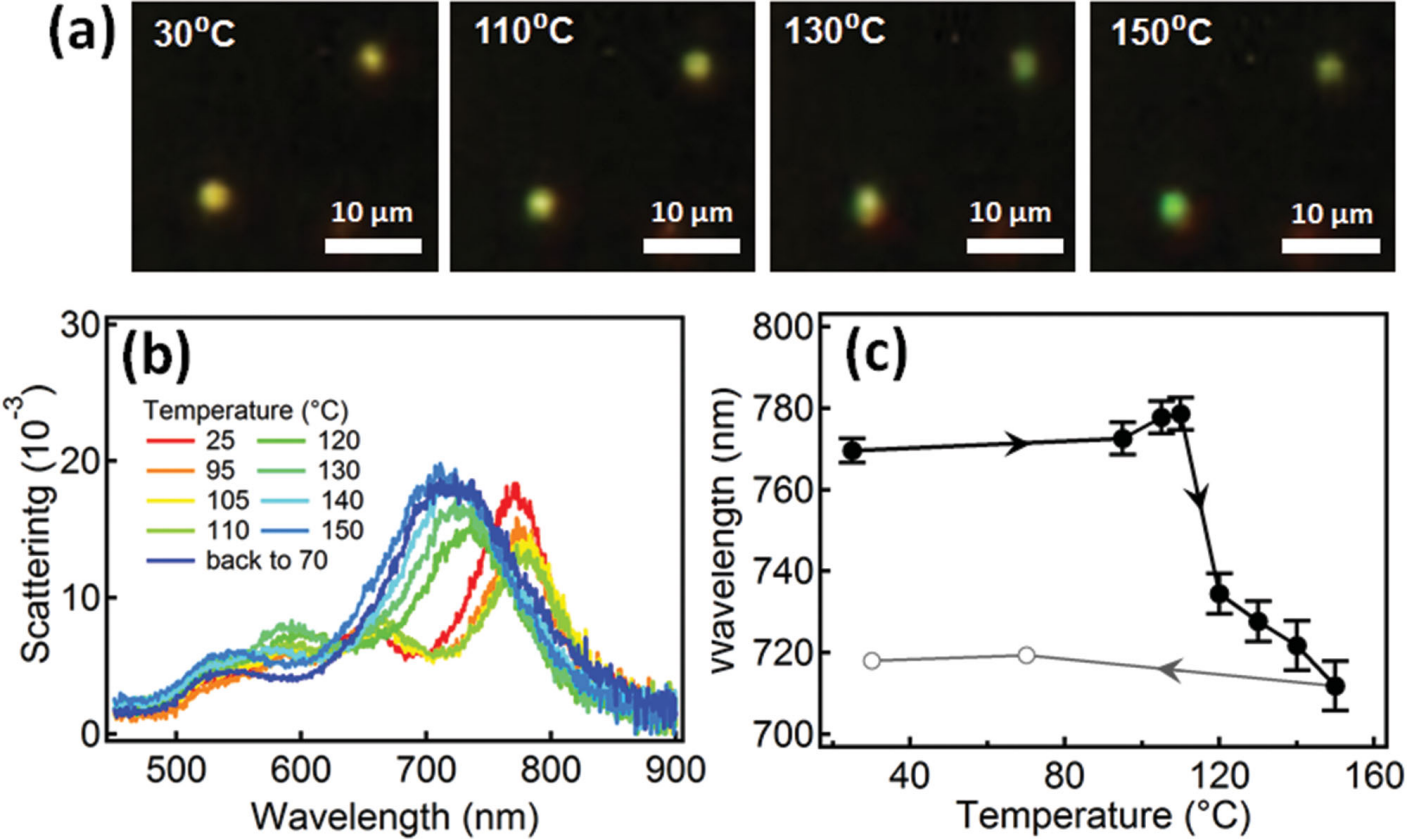
a) Dark field images of 80 nm Au NPoM with 4 nm PS spacer at increasing annealing temperatures. b) Scattering spectra at different annealing temperatures. c) Extracted resonance wavelength in one temperature cycle between 25 and 150 °C.

**Figure 4:**
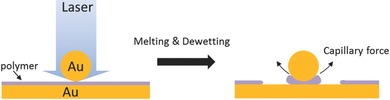
Schematic illustration of morphology changes of PS layer during laser irradiation.

We find irradiating the Au NPoM with either blue (447 nm) or red (635 nm) lasers leads to blue‐shifts of the coupled plasmons although the former produces much larger spectral shifts (150 nm) than the latter (50 nm) (Figure S6b, Supporting Information). Moreover, neither of these laser wavelengths is on resonance with the coupled plasmon, which suggests that the effect is mainly due to the direct local heating of the Au rather than plasmon‐induced heating. Irradiating individual NPoMs with different laser powers heats the PS spacer to different temperatures. We observe a linear increase of the blue‐shift only above a threshold laser power (≈200 μW) which eventually saturates (**Figure** **5**). This is predicted from the balance between capillary and van der Waals forces, since once the polymer is soft enough to flow, the meniscus forms a characteristic shape. We find similar behavior for all NPoMs on the same type of polymer.

**Figure 5:**
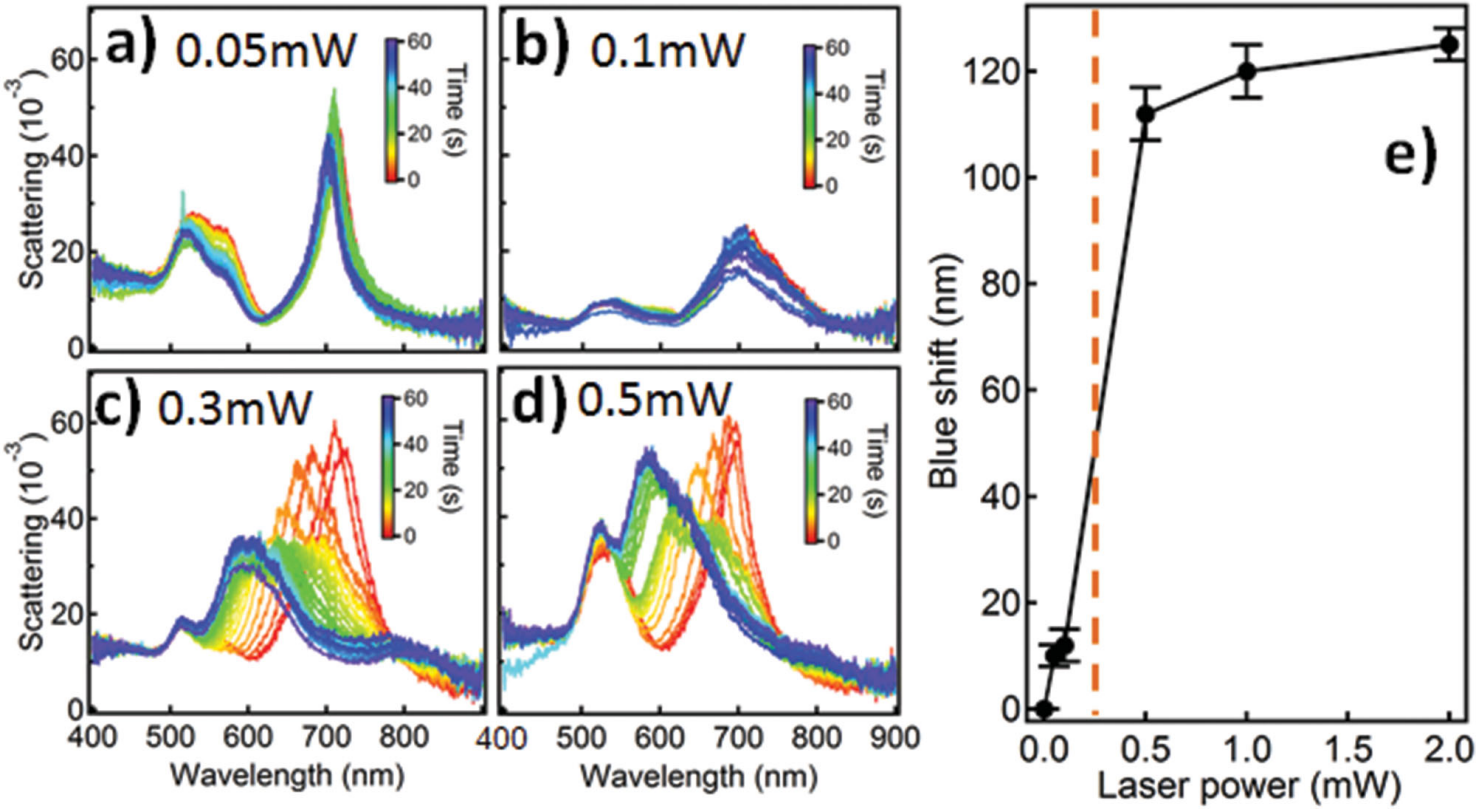
Power‐dependent irradiation of individual Au NPoMs for 60 s with a) 0.05 mW, b) 0.1 mW, c) 0.3 mW, d) 0.5 mW, using 447 nm laser. e) Extracted maximum blue‐shift versus irradiation power.

Such blue‐shifts upon irradiation should be general to any polymer type as long as they do not degrade under laser irradiation. We find that local optical melting of other polymer spacers such as PMMA (*M*_w_ = 10 000, Fluka) also causes blue‐shifts of the coupled plasmon mode (Figure S7, Supporting Information), although the observed shifts are smaller (≈50 nm). This can be attributed to differences in surface energy and viscoelastic properties of the different polymer. Our approach allows considerable freedom in optimizing the desired plasmonic tuning characteristics by harnessing polymer surface energies on the nanoscale. We note that producing such ultrathin polymeric layers may not be possible in all cases, and optimisation of this aspect is also crucial to achieve broad and selective tuning.

In conclusion, we demonstrate light‐induced tuning (by up to 150 nm) of strongly coupled plasmon resonances in the Au NPoM geometry. The ultrathin polymer spacer is softened locally via laser irradiation and bulges underneath the Au NPs through strong capillary forces. In this way Au NPs are lifted away from the Au surface and continuous blue‐shifts obtained using different irradiation durations and powers. This work allows accurate, on‐demand and remote tuning of the plasmon resonance to a desired resonance wavelength, which is significant for both fundamental understanding of the plasmon coupling in NPoM constructs and application in tunable SERS, photovoltaic, and coupling to electronic devices.

## Experimental Section

*Sample Fabrication*: The Au mirror is fabricated via e‐beam evaporation (Lesker E‐beam evaporator). A clean Si wafer (rinsed with acetone, isopropanol and deionized (DI) water and dried under nitrogen) is coated with 5 nm Ti film (0.5 Å s^−1^) as the adhesive layer, followed by depositing another 70 nm Au film (1 Å s^−1^) as the mirror. A polystyrene (*M*_p_ = 10110, *M*_w_/_Mn_ = 1.02, Polymer Laboratories) toluene solution with concentration of 0.2 mg mL^−1^ is spin‐coated (5000 rpm, 1 min) onto this Au mirror surface as the spacer layer. The thickness of the PS films formed on the Au mirror are measured by ellipsometry (α‐SE), giving estimates of 4 ± 0.5 nm. A 100 μL of Au NP solution (80 nm, BBI) is then drop‐cast onto the substrate for 5 min and rinsed with DI water to remove unabsorbed Au NPs.

*Characterizations*: A CW laser with wavelength of 447 nm is fiber‐coupled into a dark field optical microscope (BX51, Olympus) through a single mode fiber, which is confocally aligned with a detection fiber coupled to a spectrometer (QE65000, Ocean Optics). The laser spot size on the sample is 450 ± 50 nm and the power density covers the range 0.3 to 12 nW nm^−2^. Dark field images are taken with ×100 objective (NA = 0.8), and the scattering spectra recorded after every 2 s of irradiation when the laser is temporarily shuttered off. The power of the laser is adjusted up to 2 mW on the sample for irradiation. For the control experiment using global thermal heating, a Linkam heating stage was used to raise the temperature of the substrate to 150 °C at a rate of 10 °C min^−1^, and then left to cool to room temperature. The scattering spectra and dark field images of the same Au NP were taken at different temperatures. The SEM images were captured at accelerating voltage of 5 kV with a LEO 5130VP SEM (Zeiss).

*Simulation*: The numerical simulations used BEMAX, a Maxwell solver using the boundary‐element method.^23^ The dielectric functions were obtained from Johnson and Christy. The PS was modeled as a layer of refractive index *n* = 1.58 between a bulk gold surface and an 80 nm gold nanoparticle. The refractive index elsewhere was *n* = 1. A broadband plane wave source was selected for excitation incident at 58 °C with respect to the vertical axis, which represents the excitation conditions of the dark field objective irradiating around the collection cone with numerical aperture NA = 0.85 in the experiments. For each wavelength, the near‐field enhancement |E|^2^/|E_0_|^2^ was found within the gap between AuNP and Au surface. The gap width was varied between 1 and 20 nm.

## Supporting information

As a service to our authors and readers, this journal provides supporting information supplied by the authors. Such materials are peer reviewed and may be re‐organized for online delivery, but are not copy‐edited or typeset. Technical support issues arising from supporting information (other than missing files) should be addressed to the authors.

Supplementary
