# Supplementary material for: Capillary‐Force‐Assisted Optical Tuning of Coupled Plasmons
Source: Adv Mater. 2015 Sep 23;27(41):6457–61. doi: 10.1002/adma.201503292 (PMC4768643; doi:10.1002/adma.201503292)
Supplement: Supplementary file 1 — Supplementary [file ADMA-27-6457-s001.pdf]

Copyright WILEY-VCH Verlag GmbH & Co. KGaA, 69469 Weinheim, Germany, 2015.

ADVANCED MATERIALS

Supporting Information

for *Adv. Mater.,* DOI: 10.1002/adma.201503292

Capillary-Force-Assisted Optical Tuning of Coupled Plasmons

Tao Ding, Jan Mertens, Daniel O. Sigle, and Jeremy J. Baumberg**

Supporting Information

Capillary-Force-Assisted Optical Tuning of Coupled Plasmons

Tao Ding, Jan Mertens, Daniel O. Sigle, and Jeremy J. Baumberg**

Nanophotonics Centre, Cavendish Laboratory, University of Cambridge, CB3 0HE, UK
E-mails: dt413@cam.ac.uk (TD); jjb12@cam.ac.uk (JJB)

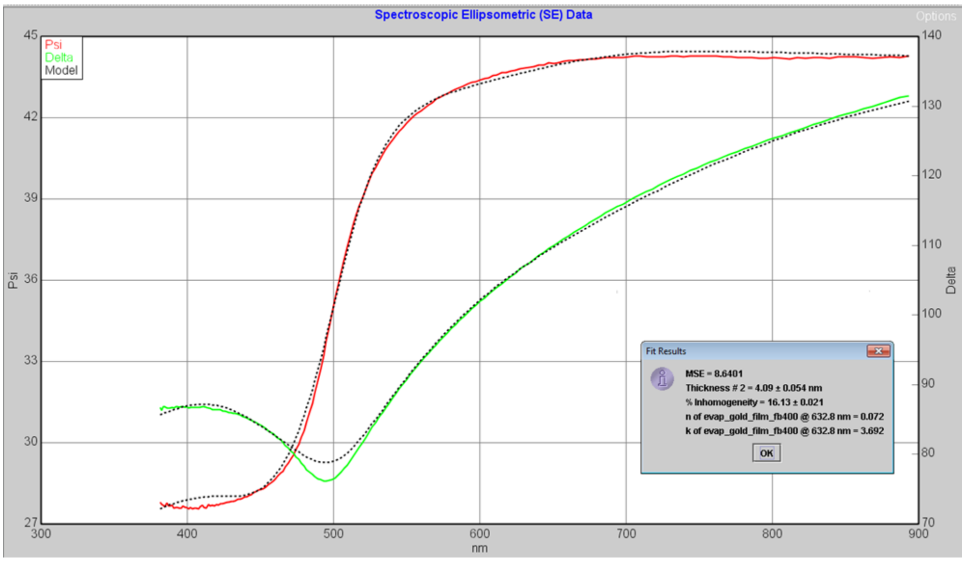

Figure S1: Spectroscopic ellipsometry measurements of the spincoated PS film on a Au substrate.

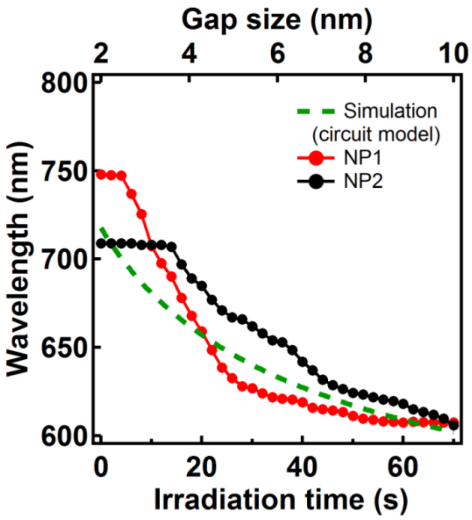

Figure S2: Longitudinal mode shift with increasing irradiation time (pumped with 447 nm 0.5 mW) of different Au NPs (red and black lines) compared to simulation using circuit model (green dash line) *vs* gap size (top axis).

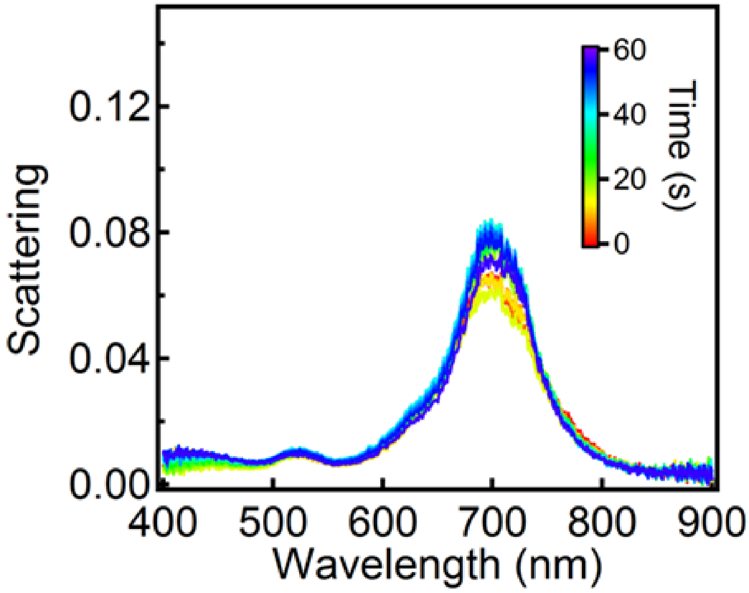

Figure S3: Irradiation of 80nm Au NPoM with evaporated 7 nm silica layer as the spacer, and irradiated under same conditions.

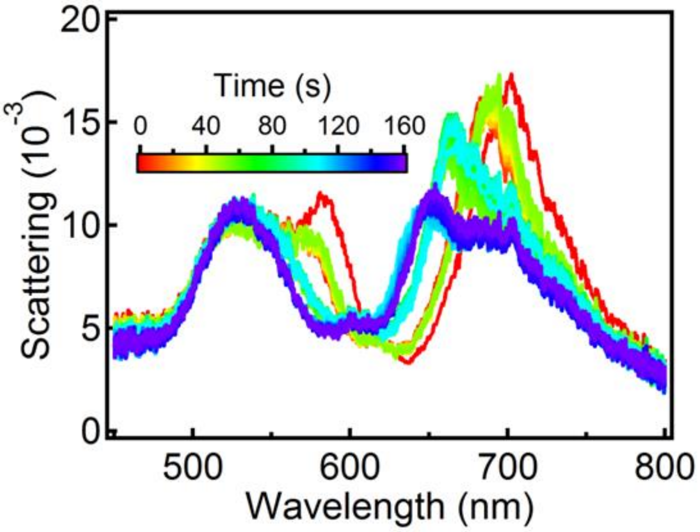

Figure S4: Evolving scattering spectra of 80 nm Au NPoM under laser irradiation (447 nm, 0.5 mW), pulsed on for 2 s and then off to allow relaxation for 40 s intervals, during which time the kinetic shift is continuously recorded. The discrete jumps on irradiation are clearly observed.

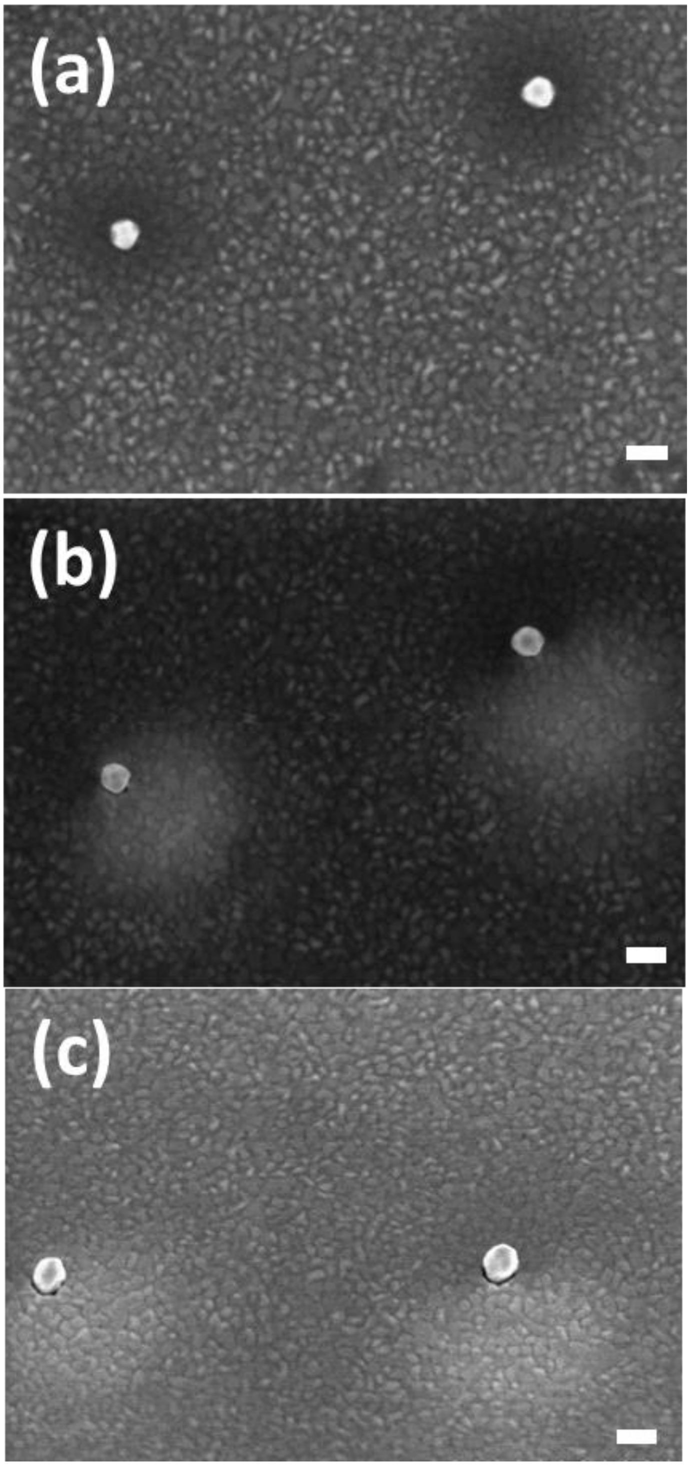

Figure S5: SEM images of Au NPoM with 4 nm PS spacer (a) before, and (b, c) after laser irradiation. (c) is 30° tilted view of the Au NPs. Scale bars are 200 nm.

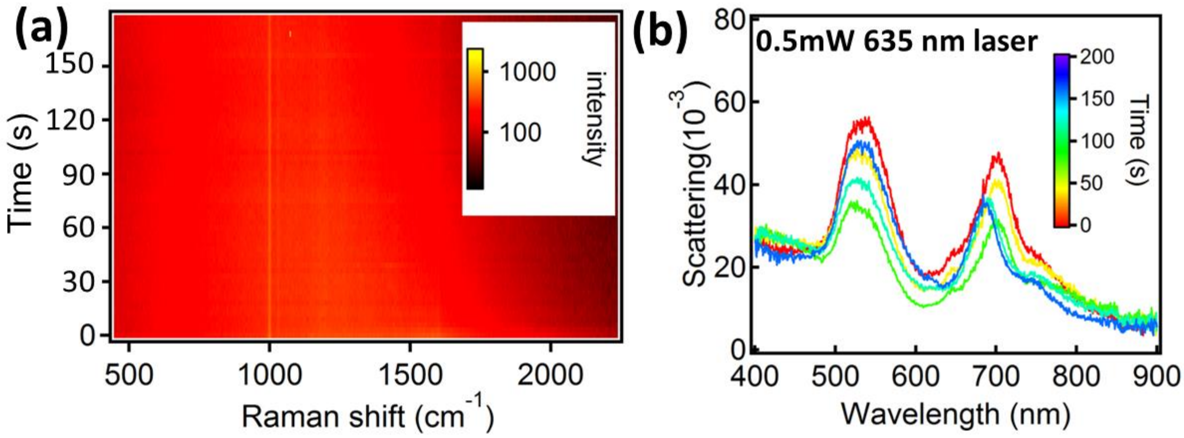

Figure S6: (a) Kinetic SERS measurement of PS within the NPoM gap, under continuous irradiation with 633 nm laser. (b) Dark field scattering spectra of Au NPoM under 635 nm laser of 0.5 mW irradiation power. A small blue shift of the longitudinal modes can be identified.

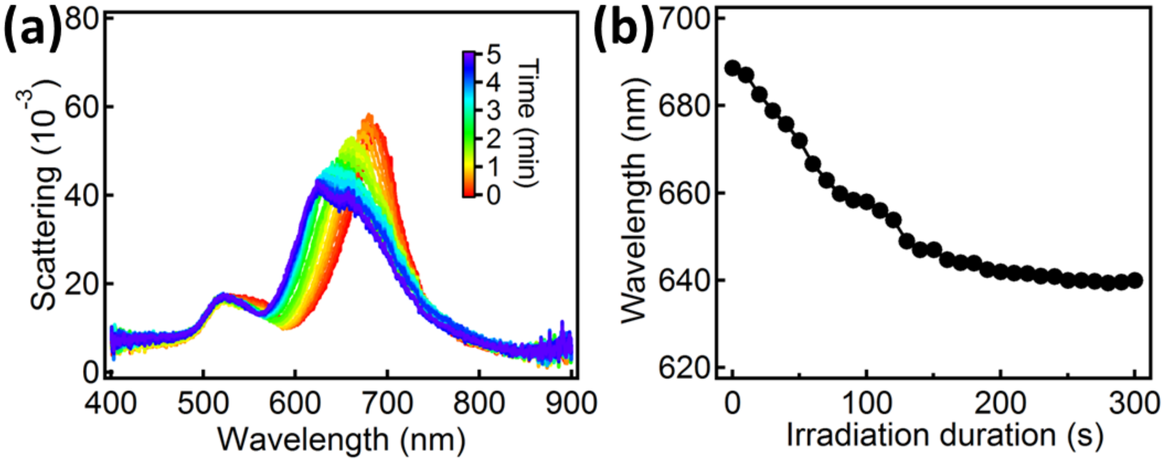

Figure S7: (a) Scattering spectra of Au NPoM using PMMA as the spacer layer, under continuous irradiation by 637 nm laser (0.5 mW). (b) Extracted evolution of wavelength of the resonant longitudinal plasmon mode with irradiation time.
